# The Emerging Role of Cardiac Conduction System Pacing as a Treatment for Heart Failure

**DOI:** 10.1007/s11897-020-00474-y

**Published:** 2020-08-28

**Authors:** Nadine Ali, Mathew Shun Shin, Zachary Whinnett

**Affiliations:** National Heart and Lung Institute, Imperial College London, Hammersmith Campus, Du Cane Road, London, W12 0HS UK

**Keywords:** Conduction system pacing, His bundle pacing, Left bundle branch pacing, Left conduction system pacing

## Abstract

**Purpose of Review:**

The aim of cardiac resynchronization therapy (CRT) is to improve cardiac function by delivering more physiological cardiac activation to patients with heart failure and conduction abnormalities. Biventricular pacing (BVP) is the most commonly used method for delivering CRT; it has been shown in large randomized controlled trials to significantly improve morbidity and mortality in patients with heart failure. However, BVP delivers only modest reductions in ventricular activation time and is only beneficial in patients with prolonged QRS duration. In this review, we explore conduction system pacing as a method for delivering more effective ventricular resynchronization and to extend pacing therapy for heart failure to patients without left bundle branch block (LBBB).

**Recent Findings:**

The aim of conduction system pacing is to provide physiological ventricular activation by directly stimulating the conduction system. Current modalities include His bundle and left conduction system pacing. His bundle pacing is the most established method; it has the potential to correct left bundle branch block and deliver more effective ventricular resynchronization than BVP. This translates into greater acute haemodynamic improvements and observational data suggests that His-CRT results in improvements in cardiac function and symptoms. AV-optimized His bundle pacing is being investigated in patients with heart failure and long PR interval without LBBB, to see if this improves exercise capacity. More recently, a technique for pacing the left bundle branch has been developed. Early studies show potential advantages including low and stable capture thresholds.

**Summary:**

Conduction system pacing can deliver more effective ventricular resynchronization than BVP, which has the potential to deliver greater improvements in cardiac function. It may also provide the opportunity to extend pacing therapy for heart failure to patients who do not have LBBB. Further data is required from randomized trials to assess these promising pacing techniques.

## Introduction

Cardiac resynchronization therapy (CRT) is as an important treatment for patients with heart failure and cardiac conduction system disease. Cardiac conduction system abnormalities, such as left bundle branch block, lead to inefficient ventricular contraction. The aim of CRT is to use pacing therapy to normalize cardiac activation in order to improve cardiac function.

Biventricular pacing is the most established method for delivering cardiac resynchronization therapy, and its use is supported by the findings from several large randomized controlled trials [1, 2]. When delivered to patients with heart failure and QRS prolongation, BVP reduces the risk of death and improves symptoms [1, 3].

However, despite the success of BVP as a treatment, there are potential reasons to explore alternative methods for delivering CRT which include the following:

1. *More effective ventricular resynchronization*: Despite biventricular pacing, morbidity and mortality remains high. In the CARE-HF trial treatment arm, mortality was 20% and 40% of patients reached the primary endpoint of death or hospitalization for a major cardiovascular event [1]. Biventricular pacing delivers only modest reductions in QRS duration, and therefore there appears to be potential to deliver greater improvements in cardiac function if more effective ventricular resynchronization can be delivered.
2. *Alternatives to LV lead placement*: The development of more advanced tools and techniques has increased success rates of LV lead implantation, but failure of LV lead placement remains a limitation of BVP. It would be useful to have alternative options for delivering CRT, which ideally could be performed during the same procedure as the BVP attempt.
3. *Extending CRT to non-LBBB patients*: BVP has only been shown to be beneficial in patients with a broad QRS, principally left bundle branch block (LBBB). There are other conduction abnormalities which also adversely affect cardiac function. These may be best corrected using alternative methods for delivering CRT.

In this review, we will discuss the potential role of cardiac conduction system pacing as an alternative method for delivering CRT and its potential to address these challenges.

## Treatment Targets for Cardiac Resynchronization Therapy

There are several different conduction system pathologies which can lead to less efficient ventricular contraction and are therefore potential treatment targets for cardiac resynchronization therapy.

### Left Bundle Branch Block

Left bundle branch block occurs in 20–30% of patients with heart failure [4, 5] and is associated with a worse prognosis compared to patients with a normal QRS duration [5, 6].

Left bundle branch block results in delayed and less coordinated left ventricular activation, which results in less efficient ventricular contraction and therefore reduced left ventricular systolic function [7]. It may also impair left ventricular filling through the following mechanisms:

- Diastolic mitral regurgitation: delayed activation results in late ventricular contraction which can result in diastolic mitral regurgitation, which leads to reduced cardiac output [8]
- Prolonged iso-volumetric contraction and relaxation times: reduces the time available for ventricular filling [8]
- Prolonged left-sided AV delay: delayed left ventricular activation may also prolong left-sided AV delay, which may also adversely affect ventricular filling.

### PR Prolongation

PR interval prolongation is also associated with an increased risk of heart failure hospitalization and mortality in patients with heart failure [9–12]. A prolonged PR interval (PR > 200 ms) is found in 15–51% of heart failure patients [13].

Prolongation of atrioventricular delay leads to reduced ventricular filling time and may also cause diastolic atrioventricular valve regurgitation. Both mechanisms reduce overall cardiac output. Reduced cardiac output may be the mechanism for the adverse outcomes observed in patients with a long PR interval.

Sub-analysis of the COMPANION trial found that patients with a prolonged PR interval received more benefit than patients with normal PR interval [12], which provides support for the concept that correcting pathologically long AV delays with pacing therapy has a beneficial effect.

### Right Bundle Branch Block

Right bundle branch block leads to delayed activation and contraction of the right ventricle relative left ventricular activation/contraction. In patients with heart failure, this type of conduction disease is associated with higher mortality [14, 15]. Although the exact mechanism is not fully understood, the inter-ventricular dyssynchrony associated with right bundle branch block may compromise left ventricular filling.

## Biventricular Pacing—the Original Method for Delivering CRT

As the beneficial effect of biventricular pacing has been observed predominantly in patients with QRS prolongation, it has been assumed that the main mechanism of action is delivered through ventricular resynchronization.

However, when BVP pacing is delivered to patients with LBBB, it produces only modest ventricular resynchronization with relatively small reductions in QRS duration [16] and ventricular activation time [17, 18]. This limited ventricular resynchronization effect is not unexpected given that BVP utilizes slow cell-cell conduction and therefore produces non-physiological ventricular activation. This attenuates the ability of BVP to correct the mechanisms through which LBBB impairs cardiac function, which raises the possibility that ventricular resynchronization may not be the only mechanism through which CRT delivers its therapeutic effect.

We used computational modelling to investigate the mechanism through which BVP delivers it beneficial effect. Our findings suggested that up to two-thirds of the improvement in stroke volume achieved with BVP is achieved by shortening AV delay (Jones et al. 2017). Conduction delay occurring with LBBB has the effect of prolonging left-sided AV delay. The potential powerful effect of AV delay optimization provides support for the concept that prolonged AV delay may be an alternative treatment target for CRT.

**Fig. 1:**
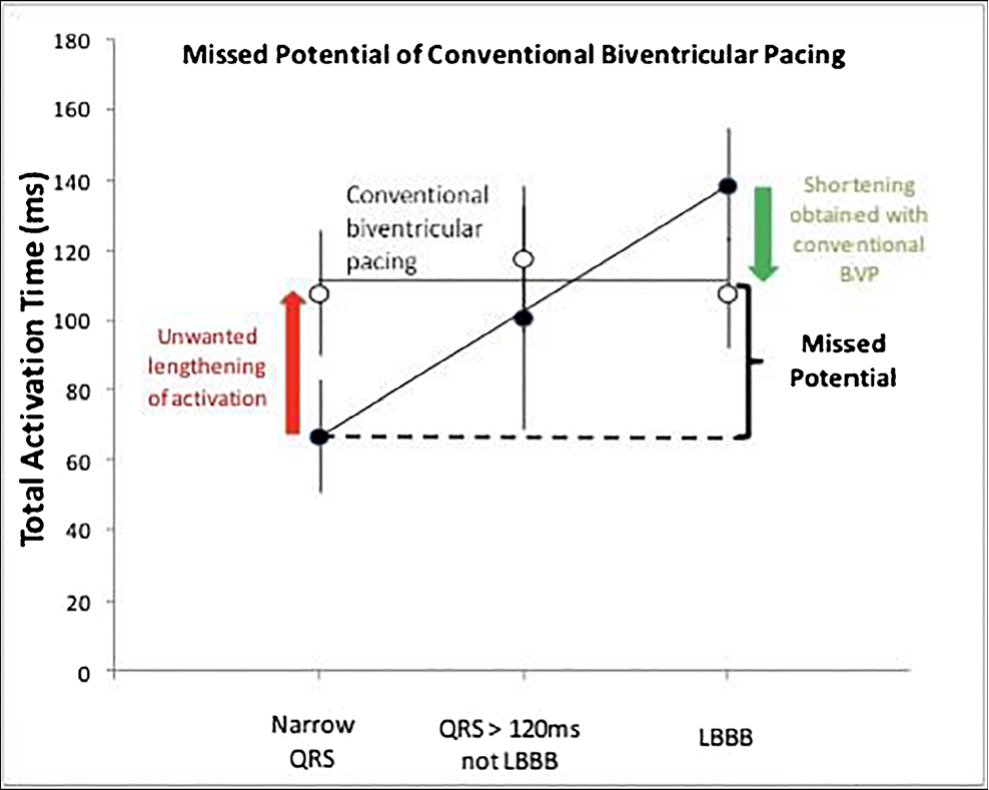
Total ventricular activation time measured using electrocardiographic imaging. This shows the change in ventricular activation time with biventricular pacing (white circles) relative baseline (black circles). In patients with a baseline narrow QRS, biventricular pacing prolongs ventricular activation time. In patients with left bundle branch block, ventricular activation time is reduced but not to physiological levels (figure from Ploux et al. [17] used with permission)

The modest ventricular resynchronization delivered with BVP implies that there is potential to deliver greater therapeutic benefit with CRT if more effective ventricular resynchronization can be delivered. Ploux et al. assessed ventricular activation time during intrinsic conduction and BVP, using non-invasive epicardial mapping (Fig. 1). They found that when BVP was delivered to patients with LBBB, ventricular activation time was shortened, but activation times did not return to the range seen in patients with an intact ventricular conduction system, which implies that there is potential to deliver more effective ventricular resynchronization.

In the computational modelling study, we found that delivering more effective ventricular resynchronization has the potential to deliver greater improvements in cardiac function [18].

Furthermore, when BVP is delivered to patients with a narrow QRS duration, this results in prolongation in ventricular activation time compared with intrinsic conduction. Therefore, biventricular pacing causes ventricular dyssynchrony when delivered to patients with an intact ventricular conduction system. This ventricular dyssynchrony can be harmful as was demonstrated in the ECHO CRT trial [19], where BVP delivered to people with a narrow QRS duration and LV impairment increased mortality by 40%. Therefore, BVP may not be the optimal pacing modality for delivering CRT to patients with non-LBBB treatment targets.

## Conduction System Pacing

### His Bundle Pacing

His bundle pacing involves direct stimulation of the cardiac conduction system, at the level of the His bundle. The concept of a pacing modality capable of preserving and potentially restoring physiological conduction is very attractive, especially in heart failure patients.

Preventing the deleterious effects of right ventricular pacing, delivering more effective cardiac resynchronization and avoiding complications associated with coronary sinus instrumentation are a few of the potential advantages.

His pacing has been shown to be technically feasible [20] and implant success has increased as a result of the development of dedicated tools for delivering the lead and increased operator experience [21].

### Left Conduction System Pacing

Direct stimulation of the left bundle can be achieved by implanting a lead within the ventricular septum via the right ventricle. This novel modality has gained widespread interest as it has potential advantages over other modalities. Left bundle branch pacing utilizes the cardiac conduction system therefore avoiding the left ventricular dyssynchrony caused by BVP. By targeting the more distal conduction system, it has the potential to correct more distal conduction system disease and early data suggests that capture thresholds are low and stable over time [22].

## Conduction System Pacing for CRT in Patients with LBBB

Conduction system pacing has the potential to deliver ventricular resynchronization to patients with LBBB. It therefore represents a potential alternative to BVP when LV lead placement is not possible via the coronary sinus and possibly also as a method for delivering more effective ventricular resynchronization.

The idea of correcting both left and right bundle branch blocks by pacing the bundle of His was established five decades ago by Narula et al. [23]. At the time, the role of abnormal cardiac conduction in worsening heart failure prognosis was not widely recognized and cardiac resynchronization therapy did not exist. The study was aimed at characterizing the physiological properties of the bundle of His after anatomical studies proposed the concept of longitudinal dissociation. Their findings suggested that left bundle branch block is often caused by conduction block within the His bundle and that it is possible to correct this with His bundle pacing.

More recently, Upadhyay et al. performed invasive mapping of the His bundle and LV septum in 72 patients with the 12-lead ECG appearance of LBBB, who were undergoing VT ablation [24••]. They found block in the proximal conduction system in 64% of patients; of these, conduction block occurred with the His bundle in 46%. His bundle pacing could restore normal conduction in all these patients. In 18%, the block occurred in the proximal left bundle. In the remaining patients with a LBBB appearance on the ECG, they found intact His-Purkinje conduction, and therefore delayed left ventricular activation was likely to be due to distal conduction tissue disease and/or myocardial disease such as myocardial uncoupling.

Therefore, conduction system pacing has the potential to correct the disordered ventricular activation occurring during left bundle branch block and represents a novel modality for cardiac resynchronization.

### His-CRT

**Fig. 2:**
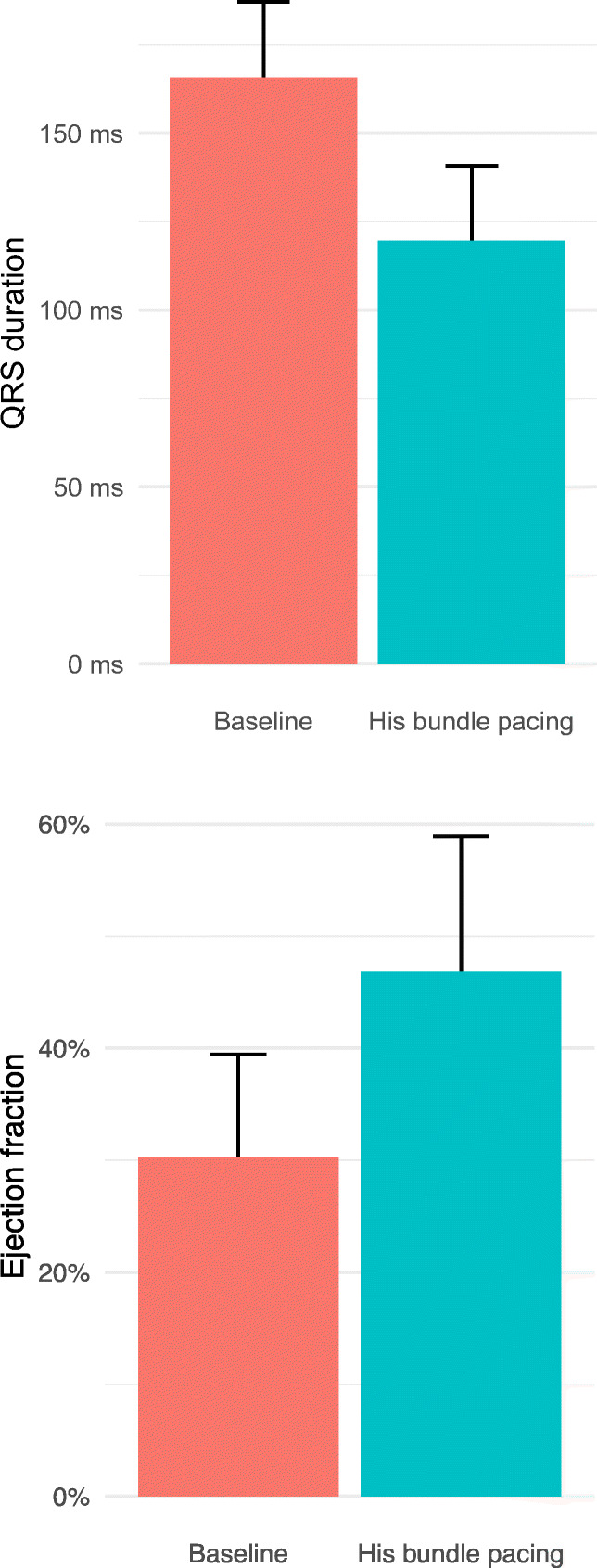
QRS shortening and left ventricular ejection fraction improvement associated with His bundle pacing, combined data from published studies (including only patients with QRS > 120 ms)

There is a growing body of evidence from observational studies, the majority of which are retrospective, that His-CRT is technically feasible (Table 1). His-CRT can deliver ventricular resynchronization with significant reductions in QRS duration compared with intrinsic conduction; the mean reduction in QRS duration observed in the combined data from multiple published studies is 49.7 ms (Fig. 2). The data from these observational studies suggest that when ventricular resynchronization is successfully delivered with His-CRT, this appears to translate into improvements in clinical outcomes such as symptoms and left ventricular contractile function. The mean improvement in left ventricular ejection fraction in these studies is 14.8%.

**Table 1:** Summary of studies of His bundle pacing as CRT in patients with abnormal cardiac conduction

Publication	Patient number*	Mean follow-up (months)	QRS (ms) baseline (mean ± SD)	QRS (ms) His bundle pacing (mean ± SD)	LVEF % baseline (mean ± SD)	LVEF % His bundle pacing (mean ± SD)
Barba-Pichardo et al. 2013 [25]	9	31	166 ± 8	97 ± 9	29 ± 5	36 ± 5
Lustgarten et al. 2015	12	6	169 ± 16	131 ± 35	26 (SD not stated)	31 (SD not stated)
Ajijola at al 2017 [26]	16	12	180 ± 23	129 ± 13	27.5 ± 10	41 ± 13
Sharma et al. 2018 [27]	44	14.4	162 ± 22	116 ± 17	28 ± 9	43 ± 13
Shan et al. 2018 [28]	5	36.2	169 ± 37	119 ± 21	35 ± 6	55 ± 8.5
Sharma et al. 2018 [29]	37	15	154 ± 24	127 ± 19	31 ± 10	39 ± 13
Huang et al. 2019 [30]	56	37	169 ± 19	114 ± 25	32 ± 9	56 ± 11
Upadhyay et al. 2019 [31•]	16	6.2	174 ± 18	125 ± 22	28 (median)	34 (median)
Combined data	195		165.7 ± 22	116 ± 23	29.7^$^	44.5^$^

*Data based on patients with QRS > 120 ms selected from each study

^$^Mean is based on available data

**Fig. 3:**
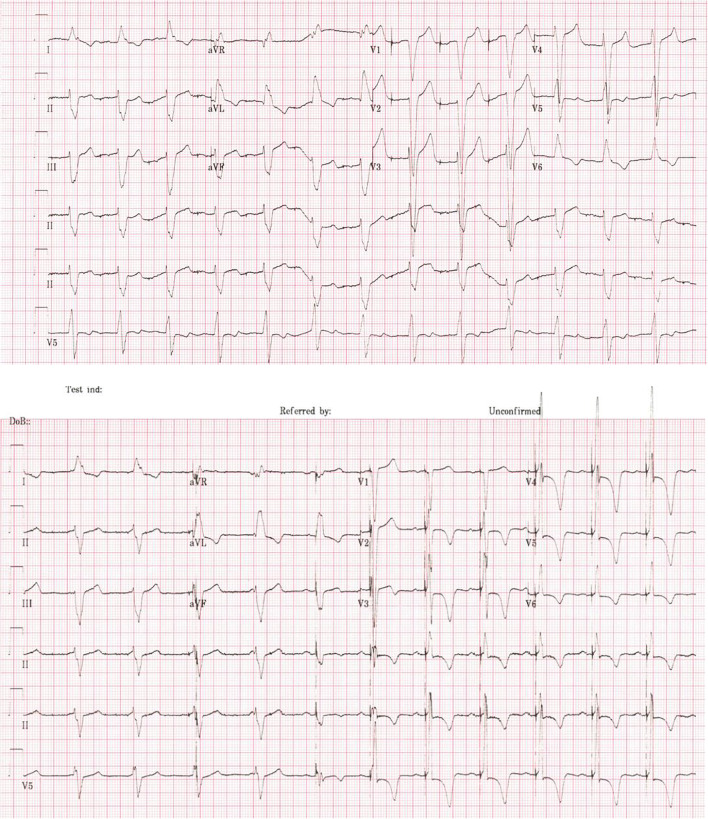
Case demonstrating His bundle pacing leading to full reversal of left bundle branch block and reduction in QRS from 170 to 105 ms. Above baseline 12-lead (atrial paced) electrocardiogram and below His bundle pacing

Figure 3 demonstrates an electrogram before and after His bundle pacing performed at our local centre. In this case, full reversal of left bundle branch block was achieved with reduction in QRS duration from 170 to 105 ms.

Therefore, these findings support the role of His-CRT as an alternative to BVP with a coronary sinus lead; however, prospective blinded endpoint data are required to confirm these findings.

### Left Conduction System Pacing-CRT

Left conduction system pacing has recently emerged as a further alternative method for delivering CRT. It has the potential advantage that by targeting the more distal conduction system, it may deliver normal physiological left ventricular activation to patients who have conduction block in the proximal left bundle rather than within the His bundle.

**Fig. 4:**
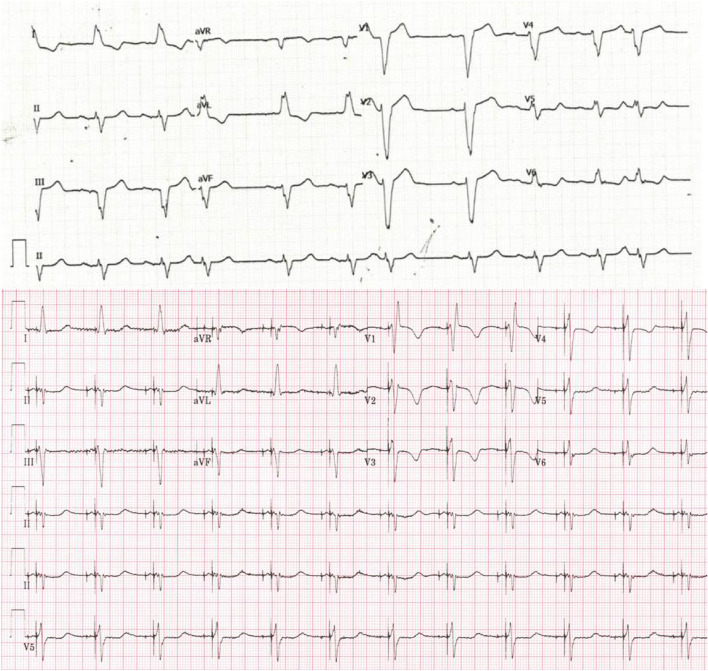
Case demonstrating left conduction system pacing. Above intrinsic 12-lead electrocardiogram and below left bundle pacing

Figure 4 demonstrates an electrocardiogram before and after left conduction system pacing in a patient with left bundle branch block.

Observational data suggests that left conduction system pacing in patients with LBBB delivers shorter QRS duration and improvements in clinical outcomes [22]. A potential disadvantage of left conduction system pacing is that it may result in non-physiological right ventricular activation, although recent studies have utilized changing the AV delay to allow fusion with intrinsic right ventricular conduction or enabling anodal capture to pace the right ventricle [32•]. It is not known whether this significantly impacts improvements in cardiac function.

Direct comparison with BVP is not yet available for left conduction system pacing and randomized trials have not yet been performed.

## His-CRT vs. BVP

While His-CRT shows promise as a method for delivering cardiac resynchronization, it must of course be compared with BVP which is supported by data from large randomized controlled trials. Data from adequately powered randomized controlled trials are not available to answer this question.

We have performed an acute study, using electrocardiographic imaging and a high-precision haemodynamic protocol to compare within-patient response to His-CRT and BVP. We found that His-CRT delivered significantly greater reductions in left ventricular activation time (43.3 ms vs. 16.7 ms, *p* value 0.01) and greater reductions in left ventricular electrical dyssynchrony and that this translated into significantly larger improvements of acute haemodynamic function. In patients in whom His-CRT successfully delivered ventricular resynchronization, it delivered a 60% greater improvement in acute haemodynamic function [33].

Adequately powered outcome trials are now required to assess whether these acute improvements translate into longer term outcomes.

To date, there have only been two randomized trials published which compare His-CRT with BVP. Both included small numbers of patients that were not designed to assess superiority.

The first was carried by Lustgarten et al. using a crossover design with patients randomized to each pacing arm for 6 months. Cardiac function and symptoms improved in both arms with no significant difference detected. However, only 12 patients completed the study, QRS narrowing with His-bundle pacing was achieved in 72% of cases [34].

More recently, Upadhyay et al. conducted the His-SYNC trial which enrolled 41 patients who were randomized to receive either His-CRT or BVP. The findings were limited by significant crossover between groups, 48% in the His-CRT arm and 26% in the BVP arm. The intention-to-treat analysis found no significant difference in echocardiographic or clinical endpoints between the two treatment groups. Treatment-received analysis found a trend towards a greater echocardiographic response in the His-CRT group (80 vs. 57% [TR], *P* = 0.14; 91% vs. 54% [PP], *P* = 0.078) [31•].

### Patient Selection for His-CRT

In the His-SYNC trial, the most frequent reason for crossing over in the His-CRT arm was failure to achieve the target QRS shortening (defined as a QRS of < 130 ms or a reduction from baseline QRS of > 20%). The majority of these patients were thought to have non-specific intraventricular conduction delay in which QRS prolongation may be due to more distal conduction system disease or myocardial uncoupling rather than discreet conduction block within the bundle of His. His-CRT would therefore not be expected to shorten ventricular activation in this situation. Differences in patient characteristics may be one of the explanations for the differences observed in acute implant success rates with His-CRT in previous studies (52% [31•]–90% [27]).

Better characterization of underlying conduction disease may help with patient selection. LBBB defined using standard 12-lead electrocardiography is known to be a heterogeneous entity as it is associated with different patterns of ventricular activation on detailed invasive electrical mapping [35].

Optimizing patient selection, by identifying patients who have conduction system block amenable to correction with His-CRT, would be useful in order to facilitate optimal recruitment for RCTs comparing His-CRT with BVP. Tung et al. found that the Strauss criteria were the most reliable 12-lead ECG criteria for identifying patients. However, specificity was only 55%. Alternative, ideally, non-invasive methods would be helpful. Non-invasive epicardial ECG mapping shows promise, and two main patterns of ventricular activation can be identified during left bundle branch block: global slow propagation and regions of apparent conduction block. In patients with regions of block, His bundle pacing was able to restore physiological activation [36].

Therefore, His-CRT shows promise as an alternative to BVP for delivering ventricular resynchronization. Larger clinical endpoint trials are required to determine whether the promising results observed in acute, observational, and pilot randomized studies translate into improvements in long-term outcomes.

## Can CRT Be Extended to Non-LBBB Patients?

### His Optimized Pacing for Patients with a Long PR Interval without LBBB?

While pacing therapy for heart failure is established in patients with LBBB, attempts to extend CRT to other groups of patients have been disappointing [19].

A potential alternative target for pacing therapy for heart failure is a prolonged PR interval. As discussed above, PR prolongation is associated with adverse outcomes. Shortening a pathologically long PR interval appears to be an important mechanism through with CRT delivers it beneficial effect in patients with LBBB [12, 18].

A prolonged PR interval can be corrected by pacing therapy with the aim of improving cardiac function, through optimization of ventricular filling. His bundle pacing, in theory, is the ideal way to deliver AV optimization since it enables AV delay to be corrected without introducing dyssynchronous ventricular activation, which has the potential to offset the beneficial effect of AV delay optimization.

We assessed the acute impact of AV-optimized His pacing using high-precision invasive systolic blood pressure measurements. Patients with systolic heart failure, a PR interval > 200 ms, without LBBB [37] were recruited. We found that AV delay optimization improved acute haemodynamics in this group with a mean increase in systolic blood pressure of 4 mmHg, which is around 60% of the effect size of biventricular pacing when it is delivered to patients with heart failure and left bundle branch block.

In the HOPE-HF trial, we are assessing whether these improvements in acute cardiac function translate into improvements in exercise capacity. The HOPE-HF trial is a multicentre, double-blinded, crossover study comparing AV-optimized His pacing with backup pacing only [38]. Recruitment was completed in July 2019 and the results are due to be reported later this year. This study is the largest prospective study of His bundle pacing with 167 participants receiving a device. It will therefore also provide useful information regarding implant success rates and His pacing parameters.

### His Pacing for RBBB?

People who have a 12-lead ECG pattern of RBBB have a higher risk of death than those with normal QRS duration and pattern [6]. It therefore represents another potential target for pacing therapy for heart failure. Results with BVP in patients with RBBB pattern are mixed [39, 40].

Biventricular pacing may not be the optimal method for delivering cardiac resynchronization to patients with RBBB, since left ventricular activation may be preserved during intrinsic conduction, in which case LV pacing via the coronary sinus may produce more dyssynchronous left activation that occurs during intrinsic conduction, although some patients with a RBBB ECG morphology may also have left conduction system disease and delayed left ventricular activation [41], which may account for the benefits observed in some studies in this group with BVP.

His bundle pacing appears to be an option for delivering ventricular resynchronization to patients with RBBB. In an observational study of 37 patients, Sharma et al. found that His pacing reduced QRS duration in patients with RBBB, with a mean reduction of 27 ms (*P* = 0.0001) [29]. Patients treated with His-CRT were found to obtain improvements in LV function and symptoms (NYHA class reduction from 2.8 to 2.0 and LVEF improved from 31 to 39%).

Prospective studies are required to confirm these promising findings.

### His Pacing Combined with AV Node Ablation

The first report of permanent His bundle pacing was in a group of patients with atrial fibrillation and left ventricular impairment who required AV node ablation to optimize ventricular rate control [20]. This study was performed prior to the development of dedicated delivery tools and as a result implant time was long. In this observational study, improvements in left ventricular function were observed [20].

AV node ablation renders patients pacing dependent and vulnerable to pacing-induced dyssynchrony which may lead to deteriorating in left ventricular function, especially in the presence of pre-existing impaired ventricular function. This outcome is more likely with right ventricular pacing but as discussed above, BVP also results in ventricular dyssynchrony in patients with a normal QRS at baseline.

The advantage of His bundle pacing in this group of patients is that it allows normal physiological ventricular activation to be preserved following AV node ablation.

Further observational studies have found His pacing to be technically feasible in the context of AV node ablation. AV node ablation can commonly be performed in the presence of a His lead without impacting on His lead capture. Improvements in left ventricular ejection fraction and NYHA class were observed with His pacing in combination with AV node ablation [42, 43].

### Right Ventricular Pacing–Induced Cardiomyopathy

Right ventricular pacing results in dyssynchronous ventricular activation, which may lead to right ventricular pacing–induced cardiomyopathy [44–49]. The risk is increased in patients with pre-existing impaired ventricular function and higher right ventricular pacing burden.

Delivering more physiological ventricular activation with conduction system pacing has the potential to protect from right ventricular pacing–induced cardiomyopathy. Observational data investigating His pacing supports this concept. Patients with a bradycardia indication for pacing were recruited into a non-randomized study taking place at two hospitals from the same healthcare provider. His pacing was attempted in 332 consecutive patients at one hospital, whereas 433 patients underwent right ventricular pacing at the other hospital. The primary endpoint of death, heart failure hospitalization or upgrade to BVP was significantly reduced in the His bundle pacing group (25% vs. 32%; HR: 0.71; 95% CI: 0.534 to 0.944; *p* = 0.02). This difference was observed primarily in patients with ventricular pacing > 20% (25% vs. 36% in RVP; HR: 0.65; 95% CI: 0.456 to 0.927; *p* = 0.02). The incidence of heart failure hospitalization was significantly reduced in patients who received His bundle pacing (12.4% vs. 17.6%; HR: 0.63; 95% CI: 0.430 to 0.931; *p* = 0.02) [50].

If these promising findings are confirmed in randomized controlled trials, then this would be expected to lead to a change in clinical practice.

No large randomized controlled trials have been performed, but based on the available observational data, the latest AHA guidelines recommend His bundle pacing (class IIb) in patients with block at the level of the AV node to preserve normal ventricular activation [51].

## Future Directions

Conduction system pacing shows considerable promise as a method for delivering pacing therapy for heart failure. However, there are still areas which require further work.

### Implant Success Rates

Implant success rates have improved with the development of dedicated tools for delivering the lead to the His bundle. In a multicentre retrospective registry, Keene et al. found that implant success rate of 81% could be achieved with enthusiastic implanters using current technology, improving to 87% with greater implant experience [21]. Fluoroscopy time and pacing thresholds were also found to improve with increasing operator experience.

However, many operators still find His pacing technically challenging. Therefore, before there is large-scale uptake of this approach, more advanced tools are likely to be required. In the early days of biventricular pacing, left ventricular lead implantation via the coronary sinus was also challenging, and the development of multiple different implant tools and dedicated leads resulted in the high implant success rates we see now. The development of techniques for targeting the more distal conduction system with left conduction system pacing is also likely to lead to improvements in implant success rates.

### Lead Reinterventions

Lead macro-displacements appear to be relatively rare with His bundle pacing.

However, lead intervention is required in ~ 7% of patients [21, 52]. While this lead intervention rate is similar to that observed with LV lead placement [53], it is desirable to reduce this reintervention rate. The main reason for reintervention is a rise in capture threshold. Improvements in lead technology and delivery may help reduce this intervention rate. The data from left conduction system pacing suggests that it is not as susceptible to rises in threshold, and therefore left conduction system may be preferred particularly for bradycardia indications.

### Randomized Studies

More prospective data is required to assess implant success rates and longer term pacing characteristics. While the results from observational and mechanistic studies with conduction system pacing have been encouraging, randomized controlled studies are now required to assess whether these findings translate in longer term clinical benefit.

## Summary

Conduction system pacing is a promising method for delivering pacing therapy for heart failure. Observational data suggests that it provides a viable alternative to BVP for delivering ventricular resynchronization to patients with LBBB. His-CRT can deliver more effective ventricular resynchronization than BVP and this translates in greater improvements in acute haemodynamic function. His-CRT also shows promise in patients with RBBB.

Randomized controlled trials are now required to assess whether these promising findings translate into improvements in longer term clinical endpoints.

AV-optimized His bundle pacing is being investigated in patients with heart failure and long PR interval, to see whether it improves exercise capacity, in a group of patients in whom BVP is not routinely recommended.

## Abbreviations

CRT: Cardiac resynchronization therapy
BVP: Biventricular pacing
LBBB: Left bundle branch block
